# Respiratory symptoms associated with a new lobe-based bronchial scoring system in an urban Chinese low-dose CT screening population

**DOI:** 10.1007/s00330-025-11712-z

**Published:** 2025-06-13

**Authors:** Zhenhui Nie, Geertruida H. de Bock, Rozemarijn Vliegenthart, Xiaofei Yang, Matthijs Oudkerk, Dirk-Jan Slebos, Zhaoxiang Ye, Maaike de Vries, Monique D. Dorrius

**Affiliations:** 1 Department of Epidemiology, University of Groningen, University Medical Center Groningen, Groningen, The Netherlands; 2 Department of Radiology, University of Groningen, University Medical Center Groningen, Groningen, The Netherlands; 3 Faculty of Medical Sciences, University of Groningen, University Medical Center Groningen, Groningen, The Netherlands; 4 Groningen Research Institute for Asthma and COPD (GRIAC), University of Groningen, University Medical Center Groningen, Groningen, The Netherlands; 5 Department of Pulmonary Diseases, University of Groningen, University Medical Center Groningen, Groningen, The Netherlands; 6 Department of Radiology, Tianjin Medical University Cancer Institute and Hospital, Tianjin, China

**Keywords:** Bronchial diseases, Computed tomography, Screening, Early diagnosis

## Abstract

**Objective:**

To develop a lobe-based bronchial scoring system in a general Chinese urban population undergoing low-dose CT (LDCT) screening and examining the association between the scores and the presence and absence of respiratory symptoms.

**Materials and methods:**

A total of 989 Chinese participants aged 40–74 from the NELCIN-B3 study underwent LDCT screening. The scoring system assessed bronchiectasis by summing up CT findings in each of the five lung lobes, including severity and extent of bronchial dilatation and airway wall thickness, as well as the presence of mucoid impaction. The modified Reiff score was used as comparison. Multivariable logistic regression analyses were performed to examine the relationship between bronchial scores and respiratory symptoms.

**Results:**

The study included 44.8% men with a median age of 62 years. Among 416 participants with bronchiectasis, mild bronchial dilatation and airway wall thickening of one generation were most common in the right lower lobe (20.7% and 15.2%, respectively). The percentage of lung lobes with bronchiectasis aligned with the modified Reiff score, showing high percentages in the right lower lobe of moderate score and of high score (92% and 90%, respectively). Multivariable analysis showed that high score was associated with wheeze (OR: 2.35; 95% CI: 1.16–4.75), especially in the upper lung region (OR: 2.25; 95% CI: 1.04–4.88), and with a lower likelihood of chest pain (OR: 0.49; 95% CI: 0.28–0.88).

**Conclusion:**

In a general Chinese urban population, over 40% of participants had bronchiectasis, mostly in the lower lung regions. Higher bronchial scores were positively associated with wheeze and negatively associated with chest pain.

**Key Points:**

***Question*** *Bronchiectasis is frequently detected on low-dose CT lung cancer screening. However, a lob-based bronchial scoring, its clinical relevance and association with respiratory symptoms are lacking.*

***Findings*** *Over 40% of participants had bronchiectasis, mostly in the lower lobes. Higher bronchial scores were associated with increased wheeze risk and reduced chest pain.*

***Clinical relevance*** *Early detection of bronchiectasis through CT-based scoring in lung cancer screening can improve assessment of respiratory health, enabling timely interventions and potentially reducing disease progression. Future research should explore the clinical implications of our new bronchial scoring system.*

**Graphical Abstract:**

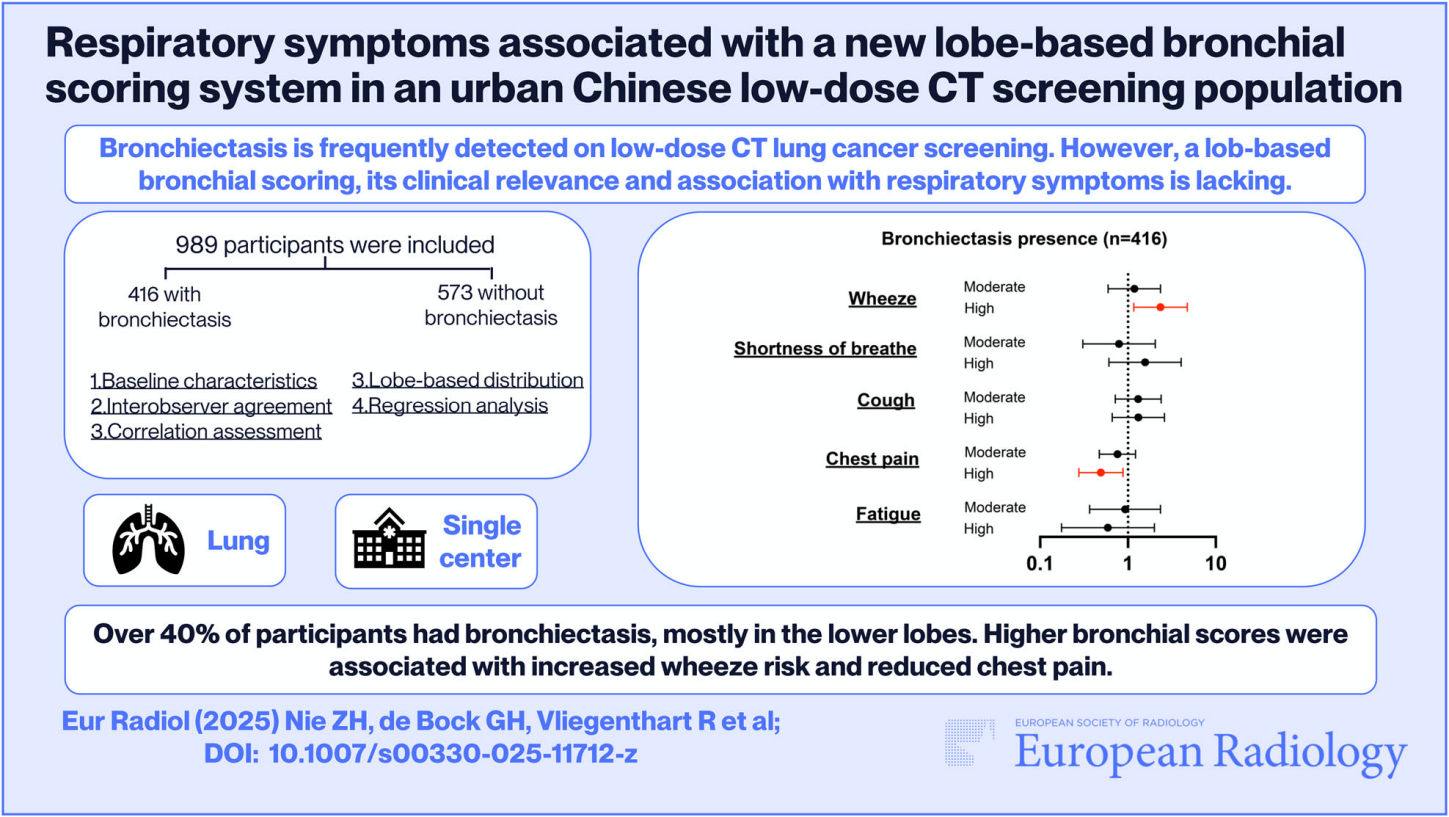

## Introduction

Bronchiectasis, an often-neglected lung disease with irreversible airway damage, is characterized by destroyed elastic and muscular tissue as a consequence of acute or chronic inflammation and infection [1, 2]. Approximately 60% of the patients report limitations in daily activities due to the disease, and on average, they experience 2–3 acute exacerbations per year [3, 4]. Except for the bronchial dilatation, the presence of mucoid impaction and airway wall thickening can cause infections or airway obstruction, ultimately leading to bronchiectasis, which is frequently identified at low-dose CT (LDCT) screening [5, 6]. Moreover, disease progression was found to be associated with both the degree of bronchial dilation and the number of bronchopulmonary segments involved [7]. To establish the diagnosis of bronchiectasis, chest CT is considered the reference standard [8, 9]. Several CT scoring systems have been developed, incorporating bronchial changes or summing lobar or segmental scores to calculate severity [10–12].

In 1991, Bhalla et al published a detailed scoring system in patients with cystic fibrosis to quantify structural bronchial abnormalities by summing scores for nine scales using thin-section CT scans [10]. Following that study, Reiff et al developed a scoring system that described the site, type, and extent of bronchiectasis using five separate scales for six lung lobes (including lingula as separate lobe) [11]. Since then, the “modified Reiff score”, defined as the product of involved lobes and three descriptors of severity, has been frequently used in studies [13, 14]. Chalmers et al proposed that bronchiectasis scoring systems can help predict progression and prognosis in bronchiectasis [12]. However, the Bhalla and Reiff scores lack consideration of specific morphological changes, such as airway wall thickening. These systems have mainly been applied to individuals with diagnosed bronchiectasis or smoking history, rather than the general population, where asymptomatic bronchiectasis is common [15, 16]. Early detection in this group is crucial for preventive assessment.

The selected populations may affect the structure of the scoring system as variations in disease prevalence, severity, and distribution could affect criteria weighting and which CT finding should be included [17]. As mostly symptomatic cohorts have been studied regarding bronchiectasis on CT, it is unknown if there is an association between radiological bronchiectasis and respiratory symptoms in a broader, general population, given the incomplete understanding of the natural course of the disease process. Therefore, in this study, we aim to develop an extensive scoring system for bronchiectasis in a general Chinese urban population and to investigate the association between the newly obtained scores and respiratory symptoms.

## Methods

### Study population

The study was approved by the ethics committee of Biomedicine Research of Second Military Medical University in China (registration number: NCT03992833), and all participants provided written informed consent. This study is based on a large Chinese population-based cohort with prospective inclusion of participants, the Nelcin-B3 study. The aim of this study was to optimize lung nodule management criteria for a Chinese population and to improve the effectiveness of CT screening for chronic obstructive pulmonary disease (COPD) and cardiovascular disease [18]. Therefore, in contrast to lung screening programs, the Neclin-B3 study not only included individuals at risk for lung cancer with a history of smoking but also included never smokers. This study selected populations from the Tianjin cohort embedded in the Nelcin-B3 study, including 4000 participants, aged 40–74, who had lived in Tianjin for at least 3 years and had no history of lung cancer [18]. Participants underwent, at baseline, a low-dose chest CT scan after written informed consent. A total of 2163 CT scans were obtained, of which 1002 consecutive scans (ID 1000–2001) were included in this study, as they underwent a comprehensive evaluation of CT findings related to bronchiectasis. In the remaining scans, CT findings were not evaluated for bronchiectasis. At the baseline screening, participants completed a questionnaire (from May to August 2017, see Supplementary Table S1) that included items assessing personal characteristics and respiratory symptoms, with a time interval of no more than 2 months from the CT examination (from June to August 2017), to identify underlying lung diseases and enhance the interpretation of CT findings.

### CT scan acquisition

All participants underwent a low-dose CT examination with a low-dose 64-detector row CT system (Somaton Definition AS 64, Siemens) at end-inspiratory breath hold [18]. The positioning of participants is head-first, supine, and with arms above the head. Chest scan acquisition used the following parameters: 120 kVp, 35 mAs, pitch of 1.0, D45F were applied to reconstruct images at 1.0/0.7 mm thickness and increment.

### Bronchial scoring system

An experienced radiologist, radiologist 1 (Z.N. with 3 years of experience in chest imaging), reviewed the LDCT scans of the Nelcin-B3 participants at lung window setting (window center: −750 HU, window width: 700 HU). The evaluation of bronchial findings was based on the D45F reconstruction kernel, utilizing the multiplanar reconstruction technique with a 1-mm thickness in the specified window setting [19, 20]. A second reader, radiologist 2 (X.Y. with 5 years of experience in chest imaging), reviewed these cases to reach interobserver agreement.

The bronchial score included bronchial dilatation, airwall thickening and mucus impaction, which was based on scales from previous scoring systems [10–12]. Bronchial dilatation was defined by abnormal widening of bronchial tree (broncho-arterial ratio > 1), along with airway morphological changes, like cylindrical, varicose and cystic type [7, 21]. Airway wall thickening was present if the thickness was at least 1 mm or 1/3 of the airway diameter [22]. Mucoid impaction referred to the filling of airways by retained secretions, which typically appears on CT as soft tissue attenuation within the airways [23]. The mere presence of these findings anywhere in the bronchial tree was considered positive for bronchial score.

Radiologist 1 determined five scales: severity and extent of bronchial dilatation, severity and extent of airway wall thickness, and the presence or absence of mucoid impaction. The severity was classified as mild, moderate and severe or significant; and the extent was classified as 1, 2 and more than 3 generations. This was done separately in five lung lobes: right upper lobe, right middle lobe, right lower lobe, left upper lobe, and left lower lobe (Table 1, Supplementary Fig. S1). The bronchial score was the sum of the above categories in each of the five lung lobes, with a maximum of 65. Bronchiectasis was defined as a total bronchial score greater than 0.

**Table 1:** Bronchial scoring system

Category	Score 0	Score 1	Score 2	Score 3
Severity of bronchial dilatation	No	Mild	Moderate	Severe
Extent of bronchial dilatation	No	1 generation	2 generations	More than 3
Severity of airway wall thickening	No	Mild	Moderate	Significant
Extent of airway wall thickening	No	1 generation	2 generations	More than 3
Mucoid impaction	No	Present	…	…

The bronchial score was the sum of the five component scores in each of the five lung lobes: (1) right upper lobe, (2) right middle lobe, (3) right lower lobe, (4) left upper lobe, and (5) left lower lobe; the maximum score was 65

For analytic purposes, the five lung lobes were aggregated into upper and lower lung regions as follows: upper lung included the right upper lobe, right middle lobe and the left upper lobe; and lower lung included the right and left lower lobes. Additionally, the right and left lung regions were defined as follows: the right lung included the right upper lobe, right middle lobe and right lower lobe; and the left lung included the left upper lobe and left lower lobe.

For comparison, we used the modified Reiff score, which was based on the number of lobes (maximum: 6, including the lingula) and three descriptors of bronchial dilatation severity (cylindrical = 1, varicose = 2, cystic = 3) [11].

To identify potential comorbidities, other LDCT findings, including emphysema and lung nodules, were documented by the senior radiologist (M.D.). Emphysema was evaluated with the presence of clearly defined or poorly defined areas of reduced attenuation or lucencies, and emphysema was defined as mild or higher which was affected at least 0.5% of a lung zone in centrilobular emphysema, one segment in panlobular emphysema, or at least 5 juxtapleural lucencies in panlobular emphysema [24]. The presence of a lung nodule was defined as all noncalcified nodules with a volume ≥ 30 mm^3^ to distinguish non-nodular lung findings [25].

### Statistical methods

All statistical analyses were conducted using SPSS 28.0 (IBM Corporation).

#### Baseline characteristics

Baseline characteristics of participants were described overall and stratified by presence and absence of bronchiectasis. Differences in participant characteristics between those 2 groups were assessed with the χ^2^ test for categorical variables and two-sample *t*-test for continuous variables.

#### Interobserver agreement

Kappa statistics for the presence of type of bronchial dilatation, and severity of airway wall thickening and mucoid impaction were calculated to assess interobserver agreement.

#### Correlation assessment

To determine the independence of individual scoring variables and the extent to which they varied together, correlations between the different CT findings with bronchiectasis were assessed using the Spearman correlation.

#### Lobe-based distribution

The total bronchial score based on the distribution in our population was subdivided into low, moderate, and high. Modified Reiff score was used as comparison, also subdivided into low, moderate and high. The bronchial score category was determined for each of the five lung lobes with bronchial score and six lung lobes with modified Reiff score, as well as each of the four lung regions (upper, lower lung regions and right, left lung regions). The percentage that represented the contribution of each lung lobe/region within each score interval was calculated by dividing the positive cases in a specific lobe/region by the total cases in that score interval. Additionally, the distribution of smoking status within each bronchial score category was analyzed.

#### Regression analysis

Multivariable logistic regression analyses were used to identify associations: (1) between the total score range and respiratory symptoms, with low score as the reference; (2) between the presence of the score per lung region and respiratory symptoms, taking the score of 0 in each lung region as reference. To assess the impact of smoking history on bronchiectasis severity, we performed multinomial logistic regression analysis with low bronchial score as the reference category. All models were adjusted for sex, age, BMI, and smoking status. Odds ratios (ORs) and 95% CIs were calculated, and *p* < 0.05 indicated a significant difference.

## Results

### Participant characteristics

**Fig. 1:**
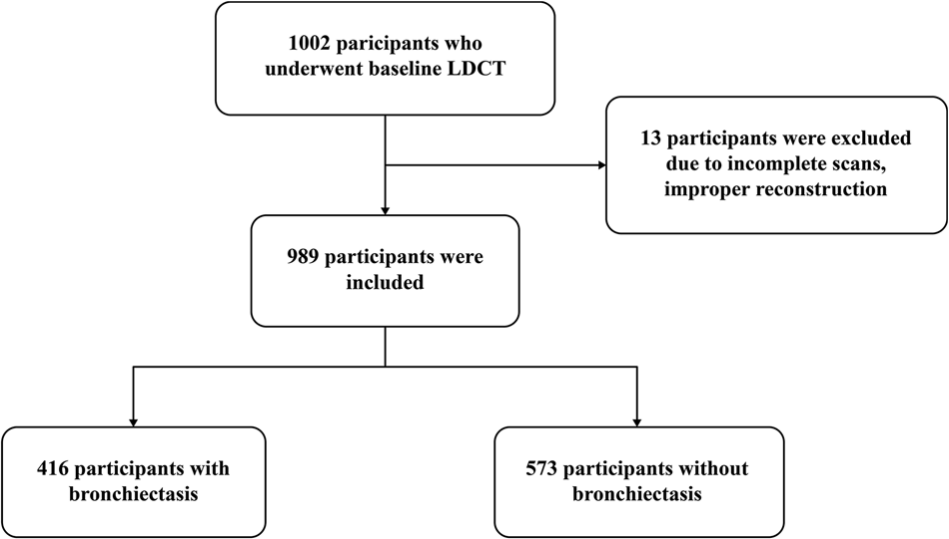
Flowchart of participant selection in this study

Of the 1002 participants, 13 cases were excluded due to incomplete CT scans, improper reconstruction of chest CT scans. Hence, 989 participants were included in our analysis (Fig. 1). The overall study population consisted of 44.8% men (Table 2) with a median age of 62 years (range 40–76). The total study population consisted of 10.4% (103 out of 989) former smokers and 23.5% (232 out of 989) current smokers, including 83.5% (328 out of 335) with more than 10 pack-years of smoking. 42.3% of participants (418 out of 989) had a BMI of 24.0–27.9 kg/m², while 40.5% (401 out of 989) had a BMI of 18.5–23.9 kg/m².

**Table 2:** Characteristics of participants, overall and stratified by the presence and absence of airway disease, defined as score 1 or more

	Bronchiectasis				
Characteristics	Total (*n* = 989)	Present (*n* = 416)	Absent (*n* = 573)		*p*-value
Demographics					
Age (median, range)	62 (40–76)	63 (42–74)	61 (40–76)		0.08
Missing	14	6	8		
Sex					**< 0.001**
Male	443 (44.8%)	238 (57.2%)	205 (35.8%)		
Female	534 (54.0%)	172 (41.3%)	362 (63.2%)		
Missing	12 (1.2%)	6 (1.4%)	6 (1.0%)		
BMI (kg/m^2^)	24.7 (14.0–43.0)	24.6 (14.0–38.0)	24.7 (15–43)		0.10
< 18.5	17 (1.7%)	10 (2.4%)	7 (1.2%)		** 0.01**
18.5–23.9	401 (40.5%)	154 (37.0%)	247 (43.1%)		
24.0–27.9	418 (42.3%)	197 (47.4%)	221 (38.6%)		
≥ 28	137 (13.9%)	48 (11.5%)	89 (15.5%)		
Missing	16 (1.1%)	7 (1.7%)	9 (1.6%)		
Education level					
Low	373 (37.7%)	146 (35.1%)	227 (39.6%)		0.15
High	616 (62.3%)	270 (64.9%)	346 (60.4%)		
Exposures					
Smoking status					**< 0.001**
No	641 (64.8%)	219 (52.6%)	422 (73.6%)		
Current	232 (23.5%)	131 (31.5%)	101 (17.6%)		
Former	103 (10.4%)	59 (14.2%)	44 (7.7%)		
Pack-years					0.88
< 10	65 (16.5%)	34 (16.3%)	31 (16.8%)		
≥ 10	270 (83.5%)	176 (83.7%)	153 (83.2%)		
Exposure					0.19
No	789 (79.8%)	340 (81.7%)	449 (78.4%)		
Yes	200 (20.2%)	76 (18.3%)	124 (21.6%)		
Clinical characteristics					
Symptoms					
Cough	190 (19.2%)	73 (17.5%)	117 (20.4%)		0.26
Wheeze	113 (11.4%)	60 (14.4%)	53 (9.2%)		** 0.01**
Shortness of breath	75 (7.6%)	29 (7.0%)	46 (8.0%)		0.54
Fatigue	51 (5.5%)	25 (6.0%)	26 (4.8%)		0.30
Chest pain	162 (26.5%)	140 (33.7%)	122 (21.3%)		**< 0.001**
Lung disease					
Asthma	14 (1.4%)	7 (1.7%)	7 (1.2%)		0.55
Emphysema	3 (0.3%)	2 (0.5%)	1 (0.2%)		0.39
Bronchitis	36 (3.6%)	17 (4.1%)	19 (3.3%)		0.52
Child respiratory diseases	95 (9.6%)	42 (10.1%)	53 (9.2%)		0.66
Tuberculosis	28 (5.0%)	12 (4.3%)	16 (5.6%)		0.48
Other	16 (1.6%)	8 (1.9%)	8 (1.4%)		0.52
Review of CT findings					
Emphysema	615 (62.5%)	309 (74.6%)	306 (53.7%)		**< 0.001**
Lung nodules	414 (41.9%)	193 (46.4%)	221 (38.6%)		** 0.01**

The definition of bronchiectasis included the CT findings of the presence of bronchial dilatation, airway wall thickening and mucoid impaction. Exposure means dust or chemical particles; other lung diseases include silicosis or pneumoconiosis. The bold number means *p* < 0.05

Among the 1002 participants, 416 (41.5%) had bronchiectasis, while 573 (57.2%) did not have bronchiectasis. Participants with bronchiectasis were, on average, older and significantly more frequently overweight, had a history of smoking, and self-reported respiratory symptoms compared with those without bronchiectasis. Furthermore, LDCT screening showed that participants with bronchiectasis had significantly more often emphysema and lung nodules (Table 2).

### Interobserver agreement

Interobserver agreement of the two radiologists was 0.79 (95% CI: 0.76–0.81) for the presence of type of bronchial dilatation, 0.91 (95% CI: 0.90–0.92) for severity of airway wall thickening and 0.93 (95% CI: 0.92–0.94) for mucoid impaction.

### Correlation between various CT findings with bronchiectasis

**Fig. 2:**
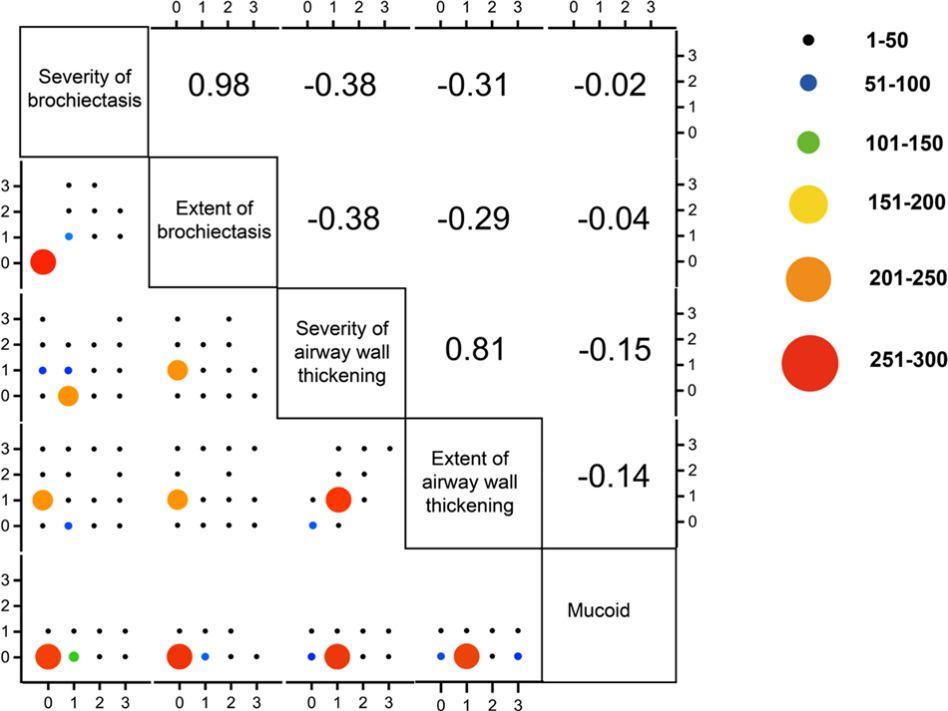
Scatterplot matrix depicting the correlations between various CT findings with bronchiectasis, along with Spearman correlation coefficients. The unit for each CT finding is listed along with the name of the finding in the diagonal axis of the figure. The X and Y axes represent scores ranging from 0 to 3 for each CT finding. The Spearman correlation coefficient is depicted on the right side of the figure. The legend bar on the right side of the figure shows different colors representing the number of various CT findings

As shown in the matrix diagram in Fig. 2, there was a strong correlation between the severity of bronchial dilatation and the extent of bronchial dilatation (*r* = 0.98, *p* < 0.001), as well as between the severity of airway wall thickening and the extent of airway wall thickening (*r* = 0.81, *p* < 0.001). There was a negative correlation between severity of bronchial dilatation and severity of airway wall thickening (*r* = −0.38, *p* < 0.001), and between extent of bronchial dilatation and extent of airway wall thickening (*r* = −0.29, *p* < 0.001).

### Lobe-based distribution of bronchial disease

Overall, the most frequent CT finding of bronchiectasis was mild bronchial dilatation and mild airway wall thickening, with mainly one generation involved (Table 3). The lower lung lobes on both sides were more frequently affected, with no difference between the left and right lung regions. Among the participants with mild bronchial dilatation, predominant lobar involvement was seen in the right lower lobe (20.7%, 86 out of 416) followed by the left lower lobe (20.5%, 85 out of 416), while the right middle lobe was the least reported (5.5%, 23 out of 416). On the other hand, a few participants (less than 10) suffered from severe bronchial dilatation, involving more than three generations, and significant airway wall thickening. The number of participants with mucoid impaction in each lung lobe was more or less comparable, except for the right middle lobe, where the number was approximately half of the other lung lobes (*n* = 7). The distribution of smoking status across bronchial score categories is shown in Supplementary Fig. S2.

**Table 3:** Lobe-based distribution of bronchiectasis

			RUL	RML	RLL	LUL	LLL	Upper lung	Lower lung	Right lung	Left lung
Bronchial dilatation	Severity	Mild	39 (9.4%)	23 (5.5%)	86 (20.7%)	25 (6.0%)	85 (20.5%)	46 (11.1%)	96 (23.1%)	98 (23.6%)	97 (23.4%)
		Moderate	4 (1.0%)	1 (0.2%)	6 (1.4%)	1 (0.2%)	4 (1.0%)	5 (1.2%)	9 (2.2%)	8 (1.9%)	3 (0.7%)
		Severe	3 (0.7%)	0 (0)	3 (0.7%)	4 (1.0%)	2 (0.5%)	6 (1.4%)	4 (1.0%)	4 (1.0%)	1 (0.2%)
	Extent	1 generation	42 (10.1%)	19 (4.6%)	63 (15.2%)	26 (6.3%)	60 (14.5%)	46 (11.1%)	69 (16.6%)	75 (18.1%)	68 (16.4%)
		2 generations	3 (0.7%)	5 (1.2%)	23 (25.5%)	3 (0.7%)	20 (4.8%)	9 (2.2%)	28 (6.7%)	25 (6.0%)	21 (5.1%)
		3 or more	1 (0.2%)	0 (0)	9 (2.2%)	1 (0.2%)	11 (2.7%)	2 (0.5%)	12 (2.9%)	10 (2.4%)	12 (2.9%)
Airway wall thickening	Severity	Mild	192 (46.3%)	91 (21.9%)	244 (58.8%)	126 (30.4%)	238 (57.3%)	197 (47.5%)	254 (61.2%)	285 (68.7%)	252 (60.7%)
		Moderate	27 (6.5%)	22 (5.3%)	37 (8.9%)	26 (6.3%)	34 (8.2%)	29 (7.0%)	40 (9.6%)	43 (10.4%)	39 (9.4%)
		Significant	1 (0.2%)	0 (0)	3 (0.7%)	0 (0)	1 (0.2%)	1 (0.2%)	3 (0.7%)	3 (0.7%)	1 (0.2%)
	Extent	1 generation	183 (44.1%)	89 (21.4%)	234 (56.4%)	122 (29.4%)	228 (54.9%)	187 (45.2%)	241 (58.1%)	265 (63.9%)	234 (56.4%)
		2 generation	11 (2.7%)	6 (1.4%)	9 (2.2%)	9 (2.2%)	7 (1.7%)	11 (2.7%)	8 (1.9%)	13 (3.1%)	10 (2.4%)
		3 or more	26 (6.3%)	17 (4.1%)	43 (10.4%)	21 (5.1%)	41 (9.9%)	29 (7.0%)	50 (12.0%)	54 (13.0%)	50 (12.0%)
Mucoid impaction	Presence	Present	15 (3.6%)	7 (1.7%)	12 (2.9%)	14 (3.4%)	15 (3.6%)	26 (6.3%)	22 (5.3%)	22 (5.3%)	24 (5.8%)

### Percentages of lung regions to bronchial score

Among the individuals with bronchiectasis (*n* = 416), the total bronchial score ranged from 1 to 32 and was categorized into low score (1–4), moderate score (5–10) and high score (11–32). As comparison, the modified Reiff score was used, which ranged from 1 to 3 and also subdivided into low (1), moderate (2) and high (3).

**Fig. 3:**
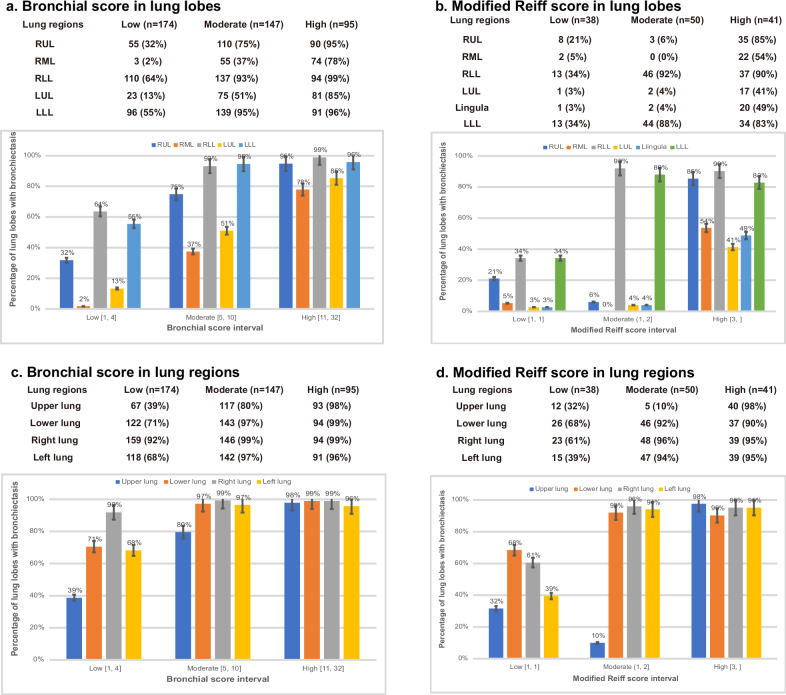
Percentages of lung lobes/regions to total score based on bronchial score and modified Reiff score separately for each score interval. **a** and **b** showed the bronchial score and modified Reiff score in lung lobes, respectively; **c** and **d** showed the bronchial score and modified Reiff score in lung regions, respectively. Bronchial score ranged from 1 to 32; subdivided into the severity of low, moderate and high. Low score was 1 to 4; moderate score was 5 to 10; high score was 11 to 32. Modified Reiff score ranged from 1 to 3; subdivided into the severity of low, moderate and high. Low score was 1; moderate score was 2; high score was 3. It is possible that more than one lobe may have positive CT findings related to the bronchial score, so that the sum of the percentages for the five lung lobes will exceed 100%

Figure 3a shows the distribution of five lung lobes to the total bronchial score. Right lower lobe and left lower lobe were the most affected for scores in the low, moderate and high score in bronchial scoring system, respectively. Consistent with the modified Reiff score (*n* = 129), significant percentages were observed in the right lower lobe of moderate score and high score (92% and 90%, respectively) (Fig. 3b). Figure 3c showed the distribution of the bronchial score separately for the upper, middle, right and left lung regions. In the upper lung, a marked increase is seen between low and moderate score (39% vs 80%), which is less evident in the lower lung (71% vs 97%). This upward trend continues in the upper lung, reaching 98% (*n* = 93) (*p* < 0.001) in the high score. However, Fig. 3d showed that the percentage of upper lung region to modified Reiff score slightly decreased to 10% (*n* = 5) (*p* = 0.01) in the moderate score.

### Bronchial score correlated with respiratory symptoms

**Fig. 4:**
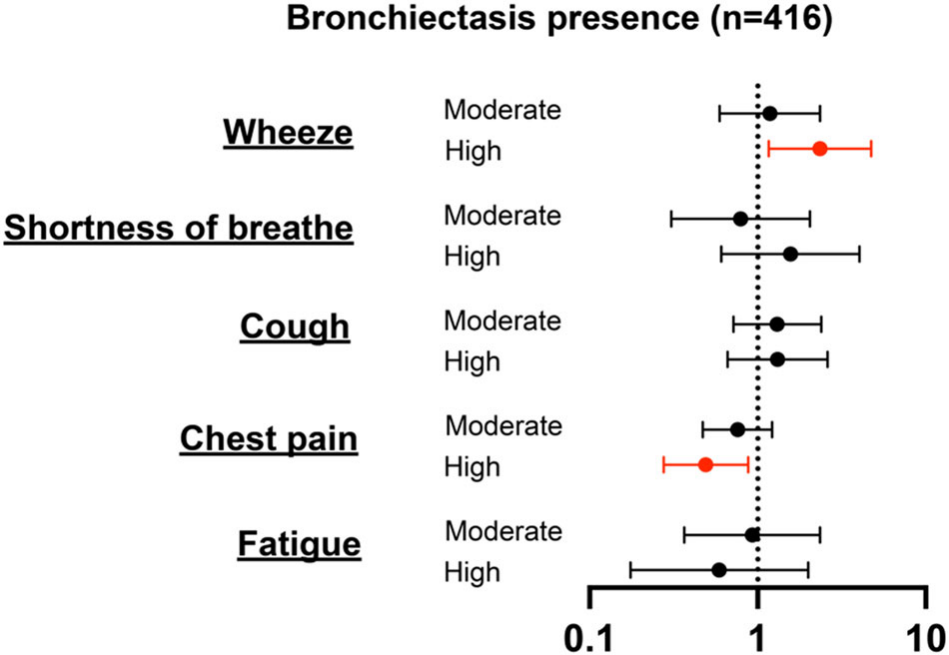
Multivariable logistic regression analyses on the association between different total bronchial score ranges and symptoms. Red means has the likelihood of increasing the prevalence of symptoms and low score was the reference. All models included age, sex, and BMI as covariates to account for potential confounding factors

Multivariable analysis showed that a high bronchial score (OR: 2.35 [1.16–4.75]) was significantly related to wheeze. In addition, high score (OR: 0.49 [0.28–0.88]) was negatively associated with chest pain. Neither moderate nor high score was associated with other respiratory symptoms, including shortness of breath, cough, and fatigue (Fig. 4, Supplementary Table S2).

**Fig. 5:**
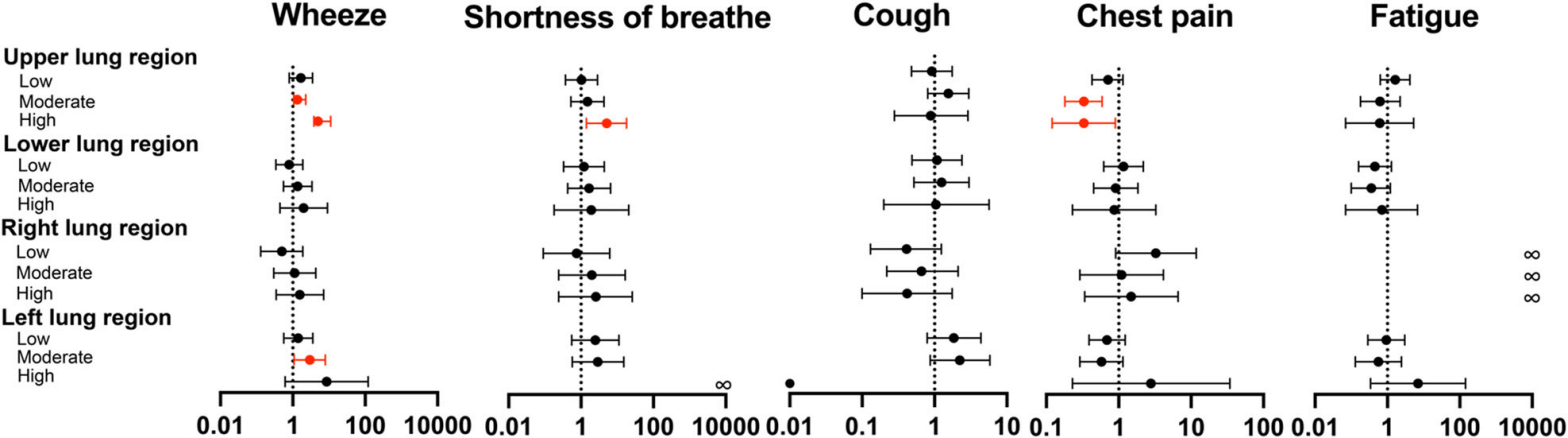
Multivariable logistic regression analyses on the association between bronchial scores of lung region and symptoms. Red means has the likelihood of increasing the prevalence of symptoms and the score of 0 in each lung region was the reference. All models included age, sex, and BMI as covariates to account for potential confounding factors

The associations with symptoms of wheeze, shortness of breath and chest pain were predominantly found in the upper lung region. In line with the overall analyses, there was no association between cough, fatigue and score interval within the different lung regions (Fig. 5, Supplementary Table S3).

Multinomial logistic regression analysis showed that current smoking status, compared to no smokers, was significantly related to moderate bronchial score (OR: 2.43 [1.26–4.68]) and high bronchial score (OR: 2.84 [1.34–6.02]) (Supplementary Table S4).

## Discussion

In this study, we aimed to establish a scoring system for semi-quantitative assessment of bronchiectasis in the general Chinese population. Of the 416 participants with CT evidence of bronchial disease in a general Chinese urban population, mild bronchial dilatation and airway wall thickening in one generation were most frequently observed. Specifically for moderate bronchial score or higher, lower lung lobes were the most affected. For high score, no regional differences were observed. The correlation between the CT findings with bronchiectasis was weak to strong. Multivariable analysis showed that high score was positively associated with wheeze, which was more apparent in the upper lung region. The association with chest pain was also predominantly found in the upper lung region; however, high score was negatively associated with chest pain.

Our study indicates that bronchiectasis was more common in the lower lung regions, probably due to gravity’s effect on the lower lobes, which facilitates mucus accumulation and leads to repeated infections and inflammation [26]. The lung regions affected by bronchiectasis often indicate a prior underlying infectious disease in the patients. It has been shown before that a lower zone distribution suggests chronic aspiration, combined variable immunodeficiency (CVID), Mounier-Kuhn syndrome, and traction bronchiectasis due to idiopathic interstitial pneumonias and asbestosis [27]. In contrast, nontuberculous mycobacterial (NTM) infection is considered a major cause of bronchiectasis, typically affecting the middle lung regions in North America [28]. However, in our study, among participants with bronchiectasis, 12 had a history of tuberculosis, with 7 showing bronchiectasis predominantly in the upper lobes, 3 in the lower lobes, and 2 in both upper and lower lobes. This pattern differs from findings in a U.S. study, which may be due to the lower incidence of NTM infection in our cohort.

To our knowledge, this is the first score that evaluates radiological bronchiectasis in a Chinese general population with no diagnosis of bronchial disease. Since our score deviates from the Reiff score, we compared the changes in the bronchiectasis score with those reported by the modified Reiff score, as the modified Reiff score is widely used in research [29]. While the modified Reiff score only records changes in the type of bronchiectasis, simplifying the assessment and saving time, it overlooks clinically relevant structural changes. These include the severity and extent of airway wall thickening and mucoid impaction, which are closely related to bronchiectasis [30]. When we compared both scores, we indeed see that using the modified Reiff score reduces the number of positive bronchiectasis cases from 416 to 129. This suggests that some positive cases may be underestimated in the modified Reiff scoring, especially in early or mild instances, where significant pathological features might be missed. While our scoring system is more comprehensive, the increase in sensitivity may detect subclinical conditions, leading to overestimation. Moreover, our scoring system will be more time-consuming for radiologists. Therefore, these results still need to be validated through further studies to confirm their clinical value. The scoring system developed in our study was primarily designed based on low-dose CT and follows the lung cancer screening protocol. Low-dose CT reduces radiation exposure while maintaining sufficient image quality to detect structural abnormalities for bronchiectasis. Although the scoring system may also be applicable to conventional-dose CT scans, validation is required to establish this, as our current dataset comes exclusively from a low-dose CT lung cancer screening program, and we do not have access to scans made with conventional-dose protocols or from other types of CT machines within the same cohort.

In the correlation assessment between the individual scoring variables, our research has found a negative correlation between bronchial dilatation and airway wall thickening, suggesting that the relationship may reflect more complex physiological or pathological processes. In the early stages of bronchiectasis, airway wall thickening may be more pronounced as it is part of the inflammatory response. However, as the disease progresses, bronchial dilatation may increase, while airway wall thickening may become less apparent because the airway wall might have been damaged or thinned [31]. In this scenario, there could be a weak or even negative correlation between airway wall thickening and bronchial dilatation. However, the number of severe dilatations in our study was limited, so we cannot confirm this. Moreover, different clinical comorbidities, such as pulmonary infections and COPD, can influence the severity and extent of CT findings with bronchiectasis [32].

Few studies elucidated the relationship between CT findings of bronchiectasis and respiratory symptoms. In our study, the lack of correlation between bronchial scores and symptoms like cough and fatigue can be due to the influence of various respiratory and systemic conditions, such as infections, asthma, chronic bronchitis, and other diseases, which may not be directly linked to bronchiectasis on CT. Additionally, individuals with early or mild bronchiectasis may not exhibit persistent symptoms. The cross-sectional design of our study also limited the assessment of symptom variations over time, potentially affecting the observed associations. Our study also found that the bronchial score was negatively associated with chest pain, especially in the upper lung regions. In clinical practice, chest pain related to bronchiectasis is often diffuse and difficult to localize accurately, typically presenting as retrosternal discomfort rather than pain confined to a specific lung region [33]. This negative correlation may be explained by disease progression: in the early stages, acute bronchial obstruction due to mucus plugging or localized lung collapse can trigger chest pain [34]. However, as bronchiectasis advances, permanent airway dilation and structural damage occur, potentially reducing airway inflammation and reactivity, which may in turn diminish the perception of chest pain. The location differs from previous findings, primarily affecting the lower lung regions [35, 36]. The question about chest pain in our questionnaire was somewhat broad, asking “Have you ever had any pain or discomfort in your chest?” and lacked specific details on the location, quality, or frequency of the pain. Additionally, our study found that wheeze and shortness of breath were positively correlated with the bronchial score, also occurring in the upper lung regions. Lesions in the upper lungs may lead to earlier and more pronounced respiratory symptoms due to their larger bronchial diameter and proximity to the tracheal origin, which directly impacts the main airflow pathway despite the gravitational effect causing mucus accumulation and infection in the lower lungs [37]. Our study found that bronchiectasis-related CT structural changes in the upper lung regions are associated with respiratory symptoms, highlighting the potential role of CT-based scoring systems in evaluating disease burden. However, given the challenges in precisely defining chest pain, further studies are needed to validate these findings and clarify their clinical significance.

Our study has several strengths. Our scoring system considers several CT findings related to bronchiectasis disease (bronchial dilatation, airway wall thickening and mucus impaction) because of its crucial role in reflecting the presence of chronic and recurrent infection resulting in bronchial inflammation, which allows for a comprehensive assessment of the disease. Additionally, this study provides information on the CT findings of bronchiectasis in the general Chinese population, distinguishing it from previous studies on individuals with bronchiectasis or those with a smoking history. This study also has some limitations. We have used cross-sectional data for our analysis, which does not allow us to determine cause-and-effect relationships between the variables or follow up on changes over time. And this study population consisted primarily of urban residents in one city in China, limiting the generalizability to other regions or those with different environmental and socioeconomic backgrounds. Second, the time interval between the CT scan and the questionnaire data collection was not in the same period, but did not exceed 2 months. We assume that the CT findings would remain stable over a short time interval. Although participants were asked to report symptoms they frequently experience, recall bias cannot be completely ruled out. Future studies could optimize the timing of data collection to ensure greater accuracy. Third, our questionnaire’s broad chest pain question, lacking details on location, quality, or frequency, may have limited the accuracy of its association with bronchiectasis. Furthermore, we did not investigate the etiology of bronchiectasis, and we also lack the pulmonary function data. Although this was not the aim of our study, a better understanding of whether bronchiectasis is associated with smoking, chronic obstructive pulmonary disease, infections, and other rare causes would be meaningful. Unfortunately, such information is incomplete in our cohort. The low prevalence of severe bronchiectasis in our study is likely due to the screening population being drawn from the general community rather than a clinical setting. While this may limit direct applicability to severe cases, it highlights the relevance of our findings for early detection and intervention in asymptomatic or mild disease stages.

A lobe-based bronchiectasis assessment scoring system can evaluate the condition of each lobe, helping doctors to gain a more detailed understanding of the distribution and severity of lesions in different lung lobes. This enables them to develop more personalized treatment plans, such as using bronchoscopy to clear secretions and administer local anti-inflammatory medication, targeting the area’s most severely affected [38]. Moreover, certain causes of bronchiectasis may more frequently affect specific lung lobes (such as chronic nontuberculous mycobacterial lung disease, which is more likely to involve the right middle lobe and lingula) [27, 39]. This can aid in the early detection and differentiation of atypical or rare causes of bronchiectasis, allowing for timely and appropriate intervention. Additionally, our findings emphasize the importance of recognizing and reporting subclinical bronchiectasis in routine clinical practice. Currently, radiologists may not consistently report mild bronchial changes, which could lead to missed opportunities for early risk stratification and patient counseling. Standardized reporting protocols may help integrate these findings into clinical decision-making.

## Conclusion

In conclusion, among Chinese participants in a lung cancer screening program, our scoring system of low-dose chest CT scans showed a positive bronchial score in over 40% of participants, mostly observed in the lower lung regions. Bronchial scores in the upper lung regions were positively associated with wheeze and shortness of breath and negatively associated with chest pain. Further research is needed to determine the clinical significance of bronchiectasis in this population and whether CT scans should be evaluated using the bronchial scoring system.

## Supplementary information

Supplementary information

## Abbreviations

BMI: Body mass index
CIs: Confidence intervals
COPD: Obstructive pulmonary disease
LDCT: Low-dose CT
ORs: Odds ratios
