# Supplementary material for: Respiratory symptoms associated with a new lobe-based bronchial scoring system in an urban Chinese low-dose CT screening population
Source: Eur Radiol. 2025 Jun 13;35(12):8140–50. doi: 10.1007/s00330-025-11712-z (PMC12634794; doi:10.1007/s00330-025-11712-z)
Supplement: Supplementary file 1 — Supplementary information [file 330_2025_11712_MOESM1_ESM.pdf]

Respiratory symptoms associated with new lobe-based bronchial scoring system in an urban Chinese low-dose CT screening population

Electronic Supplementary Material (ESM)

Eur Radiol (2025) Nie Z, de Bock GH, Vliegenthart R et al.

Supplementary Table S1. The category of participants characteristics.

Characteristics	Category	Notes
BMI	<18.5, 18.5-23.9, 24.0-27.9 and ≥ 28 kg/cm^2 [17]	-
Smoking status	Current smokers, former smokers and never smokers	Participants who smoked ⩾1 cigarette a day for ⩾ 6 months and did not quit before the interview were current smokers; any smoker who had quit before the interview were classified as former smokers; participants who had never smoked or smoked for <6 month were never smokers.
Pack years of smoking	<10 and ≥ 10 years [18]	Pack-years of smoking were calculated by multiplying the number of packs smoked per day by the number of years
Exposures	dust or chemical particles	-
Respiratory symptoms	cough (affected by weather or frequent cough when not having a cold), wheeze (frequency of occurrence), shortness of breath, fatigue and chest pain	The questions about symptoms in the questionnaire were as follows:<br>1. Cough: Does weather affect your cough? Do you often cough when you do not have a cold?<br>2. Wheeze: Do you occasionally or often have wheeze?<br>3. Shortness of breath: Do you have more signs of shortness of breath compared with others of the same age.<br>4. Fatigue: Do you have problems with performing your usual activities?<br>5. Chest pain: Have you ever had any pain or discomfort in your chest?
Lung disease history	Including known cases of tuberculosis, emphysema, asthma, chronic bronchitis, and any history of chronic respiratory diseases in childhood.	-
Other CT lung findings	emphysema, lung nodules	-

Education level	low and high [19]	Participants with a high school education or below are defined as having a low education level, while those with a college education or above are defined as having a high education level.

Supplementary Figure S1. The definition and illustration of the severity of bronchiectasis and airway wall thickening.

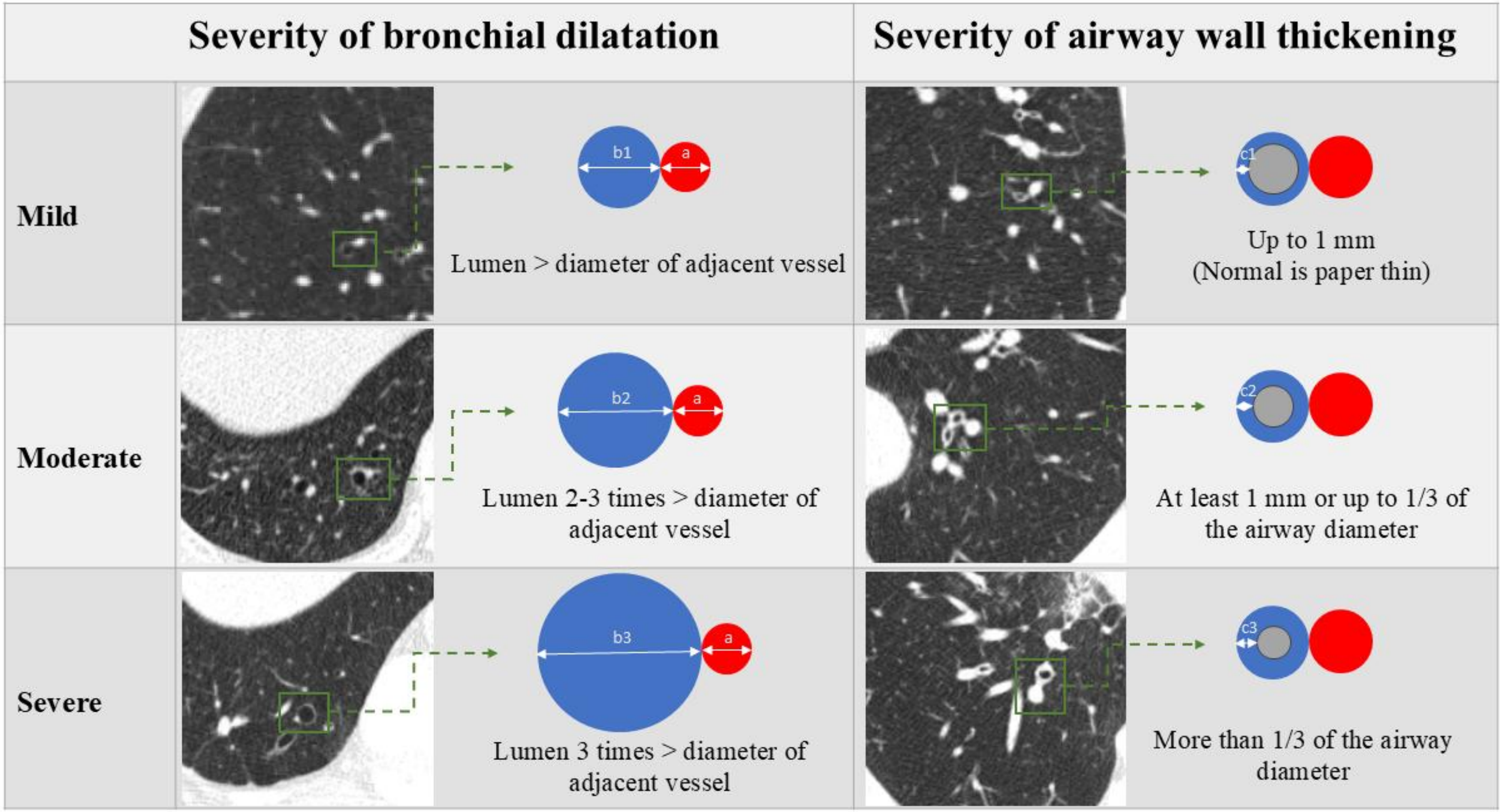

Supplementary Figure S2: Percentages of smoking status to total score based on bronchial score for each score interval. Bronchial score ranged from 1 to 32; subdivided into the severity of low, moderate and high. Low score was 1 to 4; moderate score was 5 to 10; high score was 11 to 32. The p-value is analyzed using the chi-square test.

	Low (n=174)	Moderate (n=147)	High (n=95)	*P* value
**Smoking status**				<0.001
No smokers	54%	30%	16%	
Former smokers	34%	36%	30%	
Current smokers	26%	44%	30%	

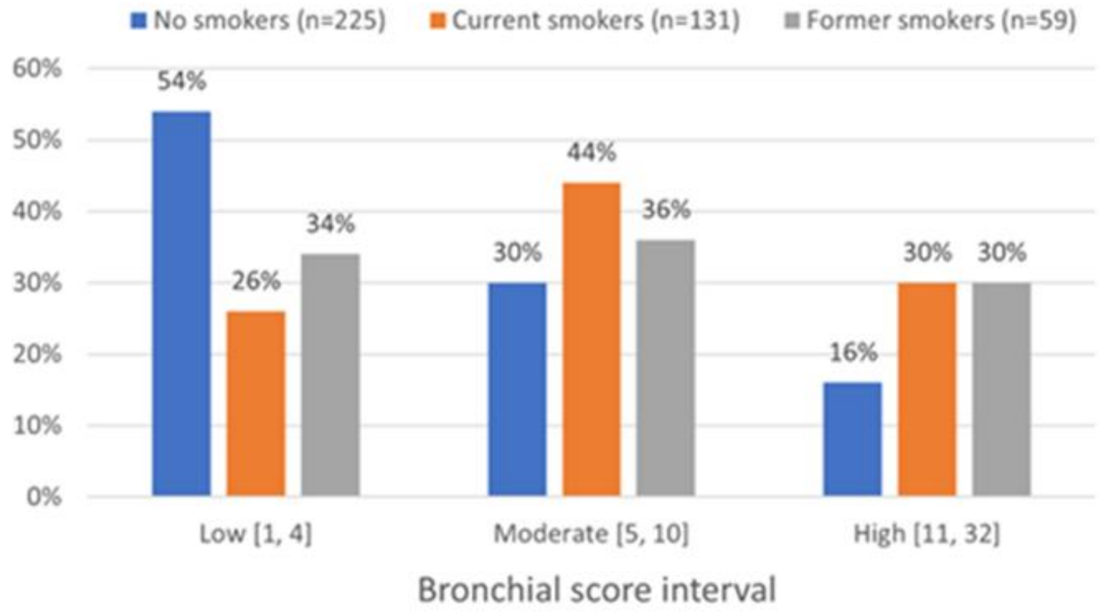

Supplementary Table S2. Odds ratios and *P* Values for the multivariate model of total bronchial scores associated with respiratory symptoms.

**Variable**	**Multivariable OR (95% CI)**	***P* value**
**Wheeze**		**0.04**
Moderate score	1.18 (0.59-2.33)	0.66
High score	2.39 (1.18-4.85)	**0.02**
**Shortness of breath**		0.38
Moderate score	0.79 (0.30-2.03)	0.62
High score	1.59 (0.62-4.10)	0.34
**Cough**		0.63
Moderate score	1.30 (0.71-2.36)	0.40
High score	1.33 (0.67-2.64)	0.42
**Chest pain**		0.06
Moderate score	0.75 (0.46-1.20)	0.23
High score	0.49 (0.28-0.88)	**0.02**
**Fatigue**		0.69
Moderate score	0.93 (0.37-2.34)	0.87
High score	0.60 (0.18-2.01)	0.40

Note: low score was the reference. All models were included age, sex, and BMI as covariates to account for potential confounding factors.

Supplementary Table S3. Odds ratios and *P* Values for the multivariate model of bronchial scores of lung region associated with respiratory symptoms

Variable	OR (95% CI) for wheeze	*P* value	OR (95% CI) for shortness of breath	*P* value	OR (95% CI) for cough	*P* value	OR (95% CI) for chest pain	*P* value	OR (95% CI) for fatigue	*P* value
**Upper lung region**		**0.05**		**0.05**		0.42		**<0.001**		0.40
Low	1.63 (0.77-3.44)	0.20	1.02 (0.37-2.81)	0.98	0.90 (0.47-1.72)	0.75	0.68 (0.42-1.12)	0.13	1.63 (0.63-4.18)	0.31
Moderate	2.25 (1.04-4.88)	**0.04**	1.48 (0.52-4.22)	0.47	1.52 (0.79-2.93)	0.21	0.31 (0.17-0.57)	**<0.001**	0.63 (0.18-2.23)	0.47
High	4.07 (1.39-11.93)	**0.01**	5.44 (1.49-19.79)	**0.01**	0.92 (0.28-2.99)	0.89	0.34 (0.13-0.93)	**0.04**	0.62 (0.07-5.42)	0.66
**Lower lung region**		0.26		0.79		0.95		0.77		0.37
Low	0.82 (0.35-1.93)	0.64	1.24 (0.34-4.49)	0.75	1.10 (0.50-2.45)	0.81	1.20 (0.64-2.26)	0.57	0.45 (0.16-1.30)	0.14
Moderate	1.39 (0.56-3.46)	0.47	1.72 (0.44-6.72)	0.44	1.28 (0.53-3.06)	0.58	0.93 (0.46-1.88)	0.85	0.36 (0.11-1.23)	0.10
High	2.36 (0.51-11.0)	0.27	2.27 (0.20-25.16)	0.51	1.19 (0.22-6.50)	0.84	1.01 (0.26-3.88)	0.99	0.75 (0.08-7.21)	0.80
**Right lung region**		**0.02**		0.09		0.20		**<0.001**		0.74
Low	0.51 (0.13-1.91)	0.31	0.76 (0.09-6.33)	0.80	0.41 (0.14-1.25)	0.12	3.32 (0.92-11.99)	0.07	∞	1.00
Moderate	1.15 (0.30-4.42)	0.84	2.02 (0.24-16.96)	0.52	0.67 (0.21-2.12)	0.50	1.11 (0.29-4.22)	0.88	∞	1.00
High	1.67 (0.37-7.59)	0.51	2.71 (0.27-27.49)	0.40	0.43 (0.10-1.83)	0.26	1.57 (0.36-6.97)	0.55	∞	1.00
**Left lung region**		**0.04**		0.63		0.41		0.30		0.38
Low	1.43 (0.56-3.62)	0.46	2.49 (0.55-11.26)	0.34	1.86 (0.79-4.40)	0.16	0.69 (0.39-1.23)	0.13	0.94 (0.29-3.00)	0.91
Moderate	3.01 (1.11-8.21)	**0.03**	3.01 (0.58-15.57)	0.19	2.29 (0.88-5.93)	0.09	0.59 (0.30-1.18)	0.40	0.56 (0.13-2.47)	0.45
High	9.02 (0.64-127.75)	0.10	∞	1.00	0	1.00	2.94 (0.24-36.51)	0.18	7.22 (0.36-146.73)	0.20

Note: the score of 0 in each lung region was the reference. All models were included age, sex, and BMI as covariates to account for potential confounding factors.

Supplementary Table S4. Odds ratios and P Values for the multinomial logistic regression model of total bronchial scores associated with smoking status.

**Outcome**	**Variable**	**Multivariable OR (95% CI)**	***P* value**
**Bronchial score**	**Smoking status**		
**Moderate**	No	1	
	Former	1.36 (0.60-3.06)	0.46
	Current	2.43 (1.26-4.68)	**0.01**
**High**	No	1	
	Former	2.12 (0.86-5.23)	0.10
	Current	2.84 (1.34-6.02)	**0.01**

Note: low bronchial score was the reference. All models were included age, sex, and BMI as covariates to account for potential confounding factors.
